# Supplementary material for: Pig Coat Color Manipulation by MC1R Gene Editing
Source: Int J Mol Sci. 2022 Sep 8;23(18):10356. doi: 10.3390/ijms231810356 (PMC9499681; doi:10.3390/ijms231810356)
Supplement: Supplementary file 1 [file ijms-23-10356-s001.zip › ijms-1878349-supplementary.pdf]

Supplemental file S1. The homologous donor sequence for *MC1R* replacement. The green uppercase letters are four SNPs corresponding to black coat color phenotype. The yellow uppercase letters in gRNA region (boxed sequence) are synonymous mutation to prevent CRISPR cleavage after HR. Red letters are *MC1R* coding region. The sequence was ligated into pTA2 Vector (TOYOBO) forming the donor plasmid which was directly used as HR template for transfection.

ggttgatgagcaggtctgagcgtggtctcacgcggcccggagggaagacaggccttcgcgggaaccgcc
cagcccacctgtgtggagagagctcgtcccctcccacctccacccccaggagcccctccttgctctcttg
gctgagaaagcactgggggcgccgagtccggctgtggcgtgtctggagccggaggcctgcccttgaagtg
gttcctggctgtgggctgtggtgaccttttgcagaggaaacgggctgtgagggaagctctggcagccacccc
gcctccgggatgagctggagcgaggtccccagcatgtgaccagggtggcccccagcagtgcgccgggt
gggggcggcggtcagggctggggccgtcctgagacagcaggcaggcaggggtgtctctgtgtcggggc
cgggagagggttggaagactcaggggcaggtccagggaagtcccgcgctccacgcctggcgtggaggc
cggcgcggggtggcgttggctgagcacaggcgaggttggccagagtgcggcggctctgcgctccaagga
gccaggaccaactgggtgccccttgcgcctcccggcaccaggcggagtgccggggctcccacggtgcgt
cccgggcccacgcccgtcacgtggccgccctcgggaggaggggctcaggcaggactttaaagatgccc
agaaagcctccgttctcctgggacctcagtcccccaccccggcctgcggcgaggcaggaagctcgaaat
gtccggacctgagcgaccgcctctccagggaagacttggtggggaggtgggctgaccacagagggtcct
ggccgggaaccgcctgggcctctctggccgggccatgcccgggcccacgttcggccagccagggcgag
tgtgggggcgcctctgggagccatgagctgagcaggaccccgagagcgacgcgccctccctgctccct
ggcgggacgatgcctgtgcttggcccggagaggaggctgctggcttccctcagctccgcgcccccagccg
ccccccgcctcgggctggccgccaaccagaccaaccagacgggcccccagtgcctggaggtgtccattc
ccgacgggctcttcctcagcctggggctggtgagcctcgtggagaacgtgctggtggtggccgccatcgcc
aagaaccgcaacctgcactcgcccatgtactacttcgtctgctgcctggccgtgtcggacctgctggtgagc
gtgagcaacAtgctggagacggccgtgctgcCgctgctggaggcgggcgccctggccgcccaggccgc
cgtggtgcagcagctggacaatgtcatggacgtgctcatctgcggctccatggtgtccTCTctGtgTttTct
Cggcgccatcgccgtggaccgctacgtgtccatcttctacgcgctgcgctaccacagcatcgtgacgctgc
cccgcgCggggcgggccatcgcggccatctgggcgggcagcgtgctctccagcaccctcttcatcgccta
ctaccaccacacggccgtcctgctgggcctcgtcagcttcttcgtggccatgctggcgctcatggcggtactg
tacgtccacatgctggcccgggcctgccagcacggccggcacatcgcccggctccacaagacgcagca
ccccacccgccagggctgcggcctcaagggcGcAgccaccctcaccatcctgctgggcgtcttcctcctct
gctgggcacccttcttcctgcacctctccctcgtcgtcctctgcccccagcaccccacctgcggctgcgtcttc
aagaacgtcaacctctttctggccctcgtcatctgcaactccatcgtggacccctcatctacgccttccgcag
ccaggagctccgcaagaccctccaggaggtgctgcagtgctcctggtgaggggggacgggcgctggag
ccaggctgcggggctgagggcagtggtgccgtcctgcggcccggttcctacgtggctgggcagccccttgg
cagagaggacgggccggacatctctgaaggtatggacgctggaccctctggggcccgacagaggaag
agccggcacttccaggaggcatggggagtgggggaggctggagagacggcggggagcgccacctcc
atccagagaccaccacgcccgcctttggggcgcgctctggggactttgcccccactggggtgggacgtgt
gcgggcagaagctgtccgggtgttgctcactgcaggacctcaggggaaggccttcgtgactgctagggaa
gcaggcgcagcgccccggcgggagggcggggcccctctcttctacggctcagtggtgaccagagggctc
ggcccatctgcaccacacacagcggcagacagcgccggccagggtccgctccggctgaagcagcggc
ctcggcctgaaccagtgaccagcgcccaggctcgggggccaggaagcagtctggtaacaaatggctgtg

ctcggcggagcccatcattcgtcctgcctctccccagaagctgcttgaagcttgagggtgggggagaggag
gtggggggtgaagagggggaaaaagtacaccaggagcctgccaggaacccagctggcctgcccagg
cgaggcagccacggccccaagtgcctgaagagccccctgggggggcgtggaggcccccgtcgcccctt
ctggatcctgtgtcagctctctctatggtgcaaaccacagccc
